# Supplementary figures and images for: Gene Analysis of Listeria monocytogenes Suspended Aggregates Induced by Ralstonia insidiosa Cell-Free Supernatants under Nutrient-Poor Environments
Source: Microorganisms. 2021 Dec 15;9(12):2591. doi: 10.3390/microorganisms9122591 (PMC8704912; doi:10.3390/microorganisms9122591)

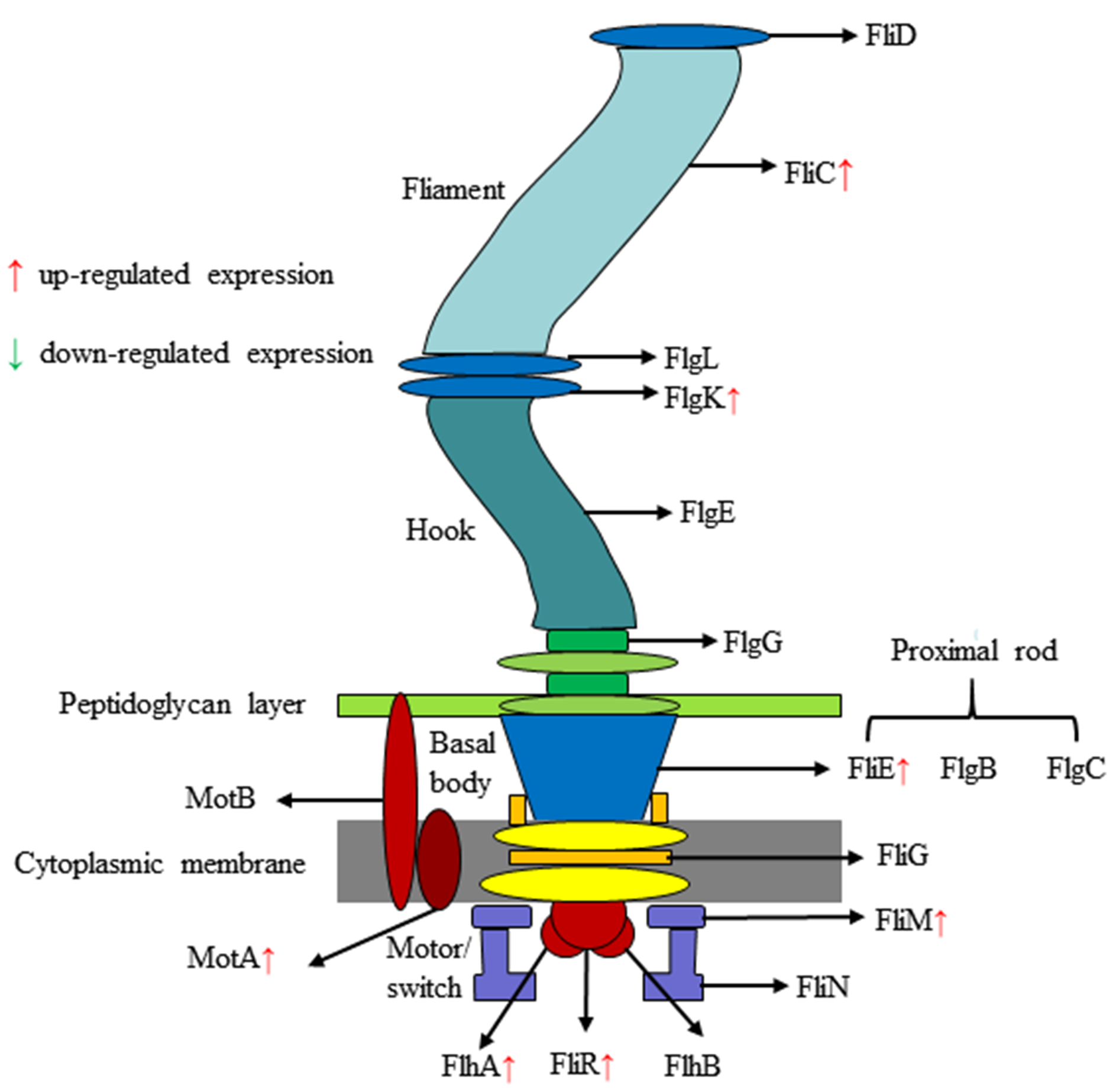

Supplement: Supplementary file 1 [file microorganisms-09-02591-s001.zip › Supplementary_Figure/Supplementary_Figure S2.tif]
